# Reliability in measurement of three-dimensional anterior pelvic plane orientation by registration with an inertial measurement unit

**DOI:** 10.3389/fsurg.2022.1011432

**Published:** 2022-11-30

**Authors:** Kyungsoo Kim, Ruoyu Wei, Yoon Hyuk Kim

**Affiliations:** ^1^ Department of Applied Mathematics, Kyung Hee University, Yongin, South Korea; ^2^ Department of Mechanical Engineering, Kyung Hee University, Yongin, South Korea; ^3^ Integrated Education Institute for Frontier Science and Technology (BK21 Four), Kyung Hee University, Yongin, South Korea

**Keywords:** anterior pelvic plane, orientation, inertial measurement units, registration, hip

## Abstract

It is strongly challenging to obtain functional movement of the pelvis based on the three-dimensional (3D) dynamic anterior pelvic plane (APP) orientation information. This study provided the 3D APP orientation measurement technique by registration with an inertial measurement unit (IMU), and its reliability was tested. The local coordinate systems of the APP and the IMU sensor were registered using two images of the pelvic part from the frontal and left sagittal views in a neutral standing posture. Then, the measurement errors in the APP orientation were analyzed by comparing the values obtained from manually measured four points in the IMU sensor and the known exact values in 10 different postures. Moreover, the errors between values obtained from manually measured three anatomical points and the known exact values were also compared. The average errors were quite small (less than 0.6°) when measuring from three anatomical points and were acceptable (1.6°–3.4°) when measuring from four points in the IMU sensor. These results indicate that the measurement of APP direction using four points in the IMU sensor could be considered reliable in terms of intra-participant and inter-participant. The present technique to register the IMU sensor position and the APP direction by taking X-ray images from the frontal and sagittal directions can be fundamental information to measure the APP direction during dynamic motion when the IMU position is obtained from the IMU sensor data instead of the four-point location information.

## Introduction

Total hip arthroplasty (THA) is a highly successful surgical intervention to restore the hip joint function and relieve pain in patients with symptomatic end-stage osteoarthritis (OA) of the hip (1). THA is also the primary treatment method for femoral neck fracture and osteonecrosis of femoral head (1). The anterior pelvic plane (APP) formed by the bilateral anterior superior iliac spines and the upper margin of the pubic symphysis was regarded as an anatomical reference for the navigation system during THA (2, 3). The pelvic tilt (PT) was defined as the angle of the APP relative to a vertical axis, and many reports denoted a certain relationship between PT and acetabular anteversion and lumbar back deformity (4, 5).

Several useful radiological-imaging techniques have been reported to obtain the APP orientation (6–12). Radiological imaging was limited to a certain posture and it merely provided the frontal and sagittal views (6–10). The computed tomography and ultrasound device can provide a three-dimensional (3D) reconstruction of the pelvic bony model to analyze the normal direction of the APP plane, but these methods easily led to APP orientation errors because of soft-tissue thickness (6–10). The EOS imaging system can provide a high-quality image and is reliable for assessing the APP orientation with lower radiation (11, 12). However, it can only provide a static posture and is also difficult to maintain in proper position while taking measurements. Therefore, a 3D pelvis-motion measurement system will be useful in clinical fields since it can resolve certain weaknesses in previous technologies.

Recently, inertial measurement units (IMUs) have been widely utilized in clinical and rehabilitation settings. The IMU is a small electric device that measures velocity and acceleration, angular velocity and acceleration, and orientation of the body using accelerometers, gyroscopes, and/or magnetometers (13–17). Functional movement of the lumbar spine was measured with the IMUs for assessment of movement-related disorders (13). The IMU-based wearable device has been used for measuring spinal shape and posture (14–16) and performing sport motion analysis (17). Moreover, the PT was analyzed using one IMU sensor (18, 19). However, it is strongly challenging to obtain functional movement of the 3D dynamic APP orientation information. This study provided the 3D APP orientation measurement technique by registration with an IMU, and its reliability was tested.

## Materials and methods

The 3D orientation of APP can be represented by two linearly independent vectors: a normal vector to APP and a vector included in the APP (20). The following procedure aims to find two vectors to represent the APP from the position information of the IMU sensor.

**Figure 1:**
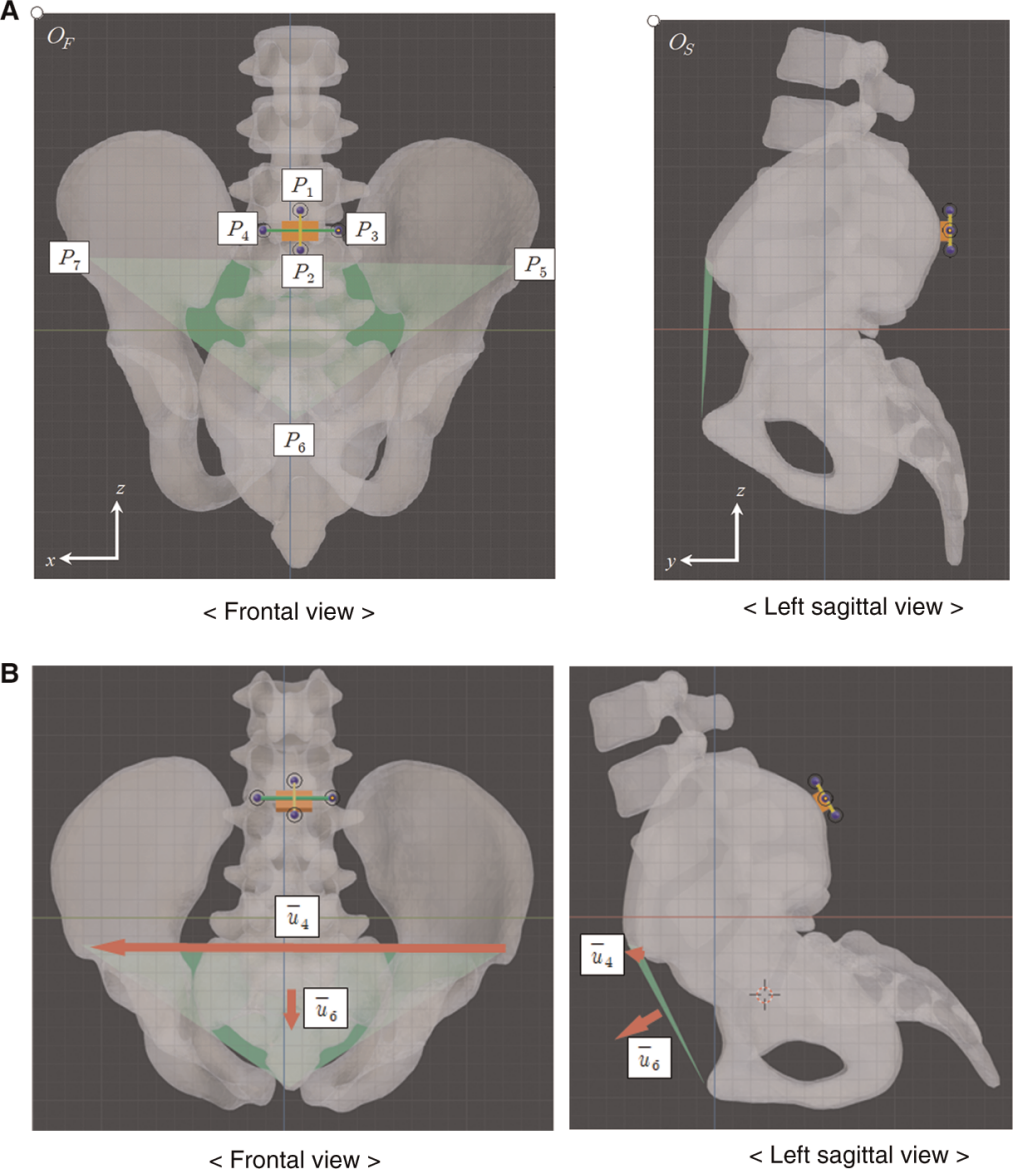
Frontal and left sagittal images of pelvic model, including the IMU sensor and the APP. (**A**) Neutral standing posture. (**B**) Flexion 30° posture.

The local coordinate system for the IMU sensor can be defined by four points (end points of the horizontal and vertical bars attached to the IMU sensor), while the local coordinate system for the APP can be defined by three points (two anterior superior iliac spine points and a marginal point on the pubic tubercles). Two images of the pelvic part from the frontal and left sagittal views in a neutral standing posture were used to register the local coordinate systems of the APP and the IMU sensor (Figure 1A). The two images were obtained by virtually projecting a 3D pelvic bony model with the IMU sensor from the frontal and left sagittal views, where the 3D pelvic model was developed in our previous study (21). Those images included the four points ($P_{1}$, $P_{2}$, $P_{3}$, and $P_{4}$) from the IMU sensor and the three points ($P_{5}$, $P_{6}$, and $P_{7}$) from the APP. Here, $P_{1}$ and $P_{2}$ were the top and bottom end points of the vertical bar, and $P_{3}$ and $P_{4}$ were the left and right end points of the horizontal bar. $P_{5}$ and $P_{7}$ were the left and right anterior superior iliac spine points, and $P_{6}$ was the marginal point on the pubic tubercles. The *x*-, *y*-, and *z*-axes in the global coordinate system were from left to right, from back to front, and from bottom to top, respectively.

In the frontal and left sagittal figures, the top left corner points were set as the origins $O_{F}$ and $O_{S}$. Next, the horizontal and vertical distances in pixel from $O_{F}$ to $P_{i}$ (1≤i≤7) were defined by $NF_{i,1}$ and $NF_{i,2}$. Similarly, the horizontal and vertical distances in pixel from $O_{S}$ to $P_{i}$ (1≤i≤7) were $NS_{i,1}$ and $NS_{i,2}$.

Let us define $v_{1}=\vec{P_{2}P_{1}}$ and $v_{2}=\vec{P_{3}P_{4}}$ for the local coordinate system of the IMU sensor and $v_{3}=\vec{P_{5}P_{7}}$ and $v_{4}=\vec{P_{5}P_{6}}$ for the local coordinate system of the APP. However, the scales of the frontal and left sagittal figures may not match. Since the real distance of $P_{1}$ and $P_{2}$ in the *z*-direction in each figure should be the same, $\mathrm{UF}=NF_{2,2}-NF_{1,2}$ and $\mathrm{US}=NS_{2,2}-NS_{1,2}$ represented the same length. By assuming the UF and US as a unit length in each figure, the vector $\vec{P_{i}P_{j}}$ can be obtained with $NF_{i,1}$, $NF_{i,2}$, $NS_{i,1}$, and $NS_{i,2}$ (1≤i≤7) as

$$\begin{array}{rl} & \vec{P_{i}P_{j}}=[\frac{NF_{i,1}-NF_{\;j,1}}{\mathrm{UF}},\frac{NS_{i,1}-NS_{\;j,1}}{\mathrm{US}},\\ & \;\;\;\frac{1}{2}(\frac{NF_{i,2}-NF_{\;j,2}}{\mathrm{UF}}+\frac{NS_{i,2}-NS_{\;j,2}}{\mathrm{US}})] \end{array}$$

where the *x*-component and *y*-component are normalized by the UF and US, respectively, and the *z*-component is the mean of *z*-components normalized by the UF and US. Then, $v_{1}=\vec{P_{2}P_{1}}$, $v_{2}=\vec{P_{3}P_{4}}$, $v_{3}=\vec{P_{5}P_{7}}$, and $v_{4}=\vec{P_{5}P_{6}}$ are represented by

$$\begin{array}{rl} & v_{1}=\vec{P_{2}P_{1}}=[\frac{NF_{2,1}-NF_{1,1}}{\mathrm{UF}},\;\frac{NS_{2,1}-NS_{1,1}}{\mathrm{US}},\\ & \;\;\;\;\frac{1}{2}\left(\frac{NF_{2,2}-NF_{1,2}}{\mathrm{UF}}+\frac{NS_{2,2}-NS_{1,2}}{\mathrm{US}}\right)] \end{array}$$

$$\begin{array}{rl} v_{2}=\vec{P_{3}P_{4}} & =[\frac{NF_{3,1}-NF_{4,1}}{\mathrm{UF}},\;\frac{NS_{3,1}-NS_{4,1}}{\mathrm{US}},\;\\ & \frac{1}{2}\left(\frac{NF_{3,2}-NF_{4,2}}{\mathrm{UF}}+\frac{NS_{3,2}-NS_{4,2}}{\mathrm{US}}\right)] \end{array}$$

$$\begin{array}{rl} v_{3}=\vec{P_{5}P_{7}} & =[\frac{NF_{5,1}-NF_{7,1}}{\mathrm{UF}},\;\frac{NS_{5,1}-NS_{7,1}}{\mathrm{US}},\\ & \;\frac{1}{2}\left(\frac{NF_{5,2}-NF_{7,2}}{\mathrm{UF}}+\frac{NS_{5,2}-NS_{7,2}}{\mathrm{US}}\right)] \end{array}$$

$$\begin{array}{rl} v_{4}=\vec{P_{5}P_{6}} & =[\frac{NF_{5,1}-NF_{6,1}}{\mathrm{UF}},\;\frac{NS_{5,1}-NS_{6,1}}{\mathrm{US}},\\ & \;\frac{1}{2}\left(\frac{NF_{5,2}-NF_{6,2}}{\mathrm{UF}}+\frac{NS_{5,2}-NS_{6,2}}{\mathrm{US}}\right)] \end{array}$$

Three unit vectors, $u_{1}=v_{1}/|\left|v_{1}\right||$, $u_{2}=v_{2}/|\left|v_{2}\right||$, and $u_{3}=u_{1}\times u_{2}$, construct an orthonormal basis for the local coordinate system of the IMU sensor. Let the 3×3 matrix $U_{0}$ be $U_{0}=[u_{1}\vdots u_{2}\vdots u_{3}]$. Moreover, another three unit vectors, $u_{4}=v_{3}/|\left|v_{3}\right||$, $u_{5}=v_{4}/|\left|v_{4}\right||$, and $u_{6}=u_{4}\times u_{5}$, represent the APP orientation, where $u_{6}$ is the normal vector to the APP, and $u_{4}$ is a vector included in the APP from left to right anatomical points. Two vectors, $u_{6}$ and $u_{4}$, are used as reference unit vectors to estimate the APP orientation in other postures.

**Figure 2:**
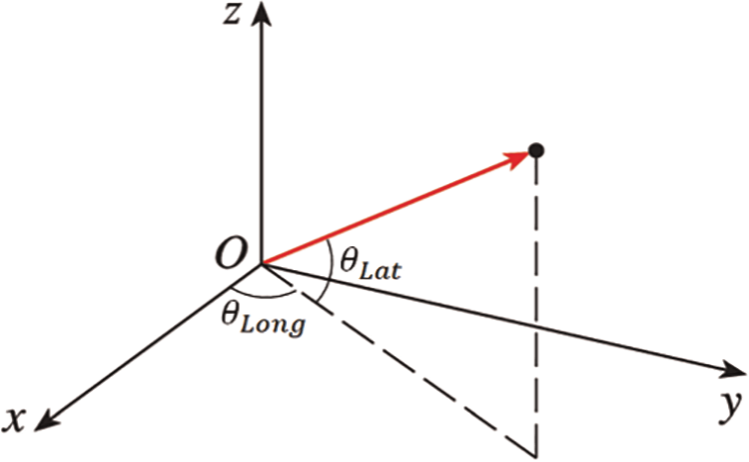
Definition of the latitude and longitude of a vector.

To geometrically understand $u_{6}$ and $u_{4}$, the latitude and longitude concepts are introduced. The latitude $\theta_{Lat}$ of a vector is defined as an angle between the vector and the *xy*-plane, where the +*z* direction is +90° and the –*z* direction is –90°. The longitude $\theta_{Long}$ of a vector is defined as an angle between the vector and *zx*-plane, where the *x*-direction is 0° and the *y* direction is 90° (Figure 2).

In an arbitrary posture, the normal vector to the APP and the vector from the left to right anatomical points can be estimated when frontal and left sagittal figures are given, only including the four end points in bars attached to the IMU sensor. Let the four points be ${\bar{P}}_{1}$, ${\bar{P}}_{2}$, ${\bar{P}}_{3}$, and ${\bar{P}}_{4}$. As in the neutral standing posture, three unit vectors, ${\bar{u}}_{1}$, ${\bar{u}}_{2}$, and ${\bar{u}}_{3}$, can be obtained and construct an orthonormal basis for the local coordinate system of the IMU sensor in an arbitrary posture. Let the 3×3 matrix *U* be $U=[{\bar{u}}_{1}\vdots {\bar{u}}_{2}\vdots {\bar{u}}_{3}]$. Under the assumption that the IMU sensor and the APP were attached to the pelvis, the normal vector to the APP ${\bar{u}}_{6}$ and the vector from left to right anatomical point ${\bar{u}}_{4}$ in the arbitrary posture can be estimated as ${\bar{u}}_{6}=UU_{0}^{-1}u_{6}$ and ${\bar{u}}_{4}=UU_{0}^{-1}u_{4}$ (Figure 1B). Let $\alpha_{Lat}$ and $\alpha_{Long}$ be the latitude and longitude of ${\bar{u}}_{6}$, and $\beta_{Lat}$, and $\beta_{Long}$ be the latitude and longitude of ${\bar{u}}_{4}$.

Two vectors, ${\bar{u}}_{6}$ and ${\bar{u}}_{4}$, also can be obtained using ${\bar{P}}_{5}$, ${\bar{P}}_{6}$, and ${\bar{P}}_{7}$, which are three anatomical points in the arbitrary posture. Two calculation methods should result in the same vectors based on the fundamental linear algebra. However, there may be measurement errors when obtaining $P_{i}$ and ${\bar{P}}_{i}$ (1≤i≤7) since the pixel values of points are manually measured.

**Figure 3:**
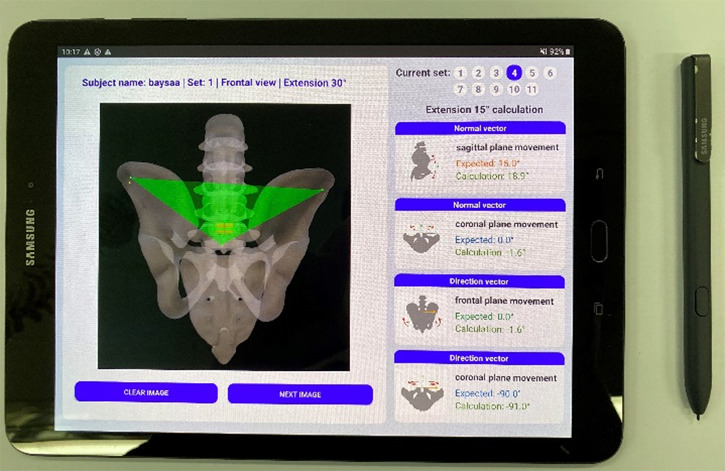
Android tablet/pencil used in the experiment.

To investigate the reliability in measuring the 3D APP orientation only using positional information from one IMU sensor, 10 volunteers (26.1 ± 3.5 years old, visual acuity 0.86 ± 0.45) participated in the test experiment with the written informed consent. An Android tablet (Samsung Galaxy Tab S3 9.7, Korea) was used to collect point data and touch pencil was used to mark the four points in the IMU sensors and the three anatomical points (Figure 3). Each participant clicked seven points on frontal and left sagittal images from eleven different image sets and repeated the experiment five times with randomly reordered image sets.

The 11 image sets were obtained using the 3D pelvic bony model: (1) neutral standing posture, (2) flexion 15°, (3) extension 15°, (4) extension 30°, (5) right rotation 15°, (6) right rotation 30°, (7) left bending 15°, (8) left bending 30°, (9) extension 15° and right rotation 15°, (10) right rotation 15° and left bending 15°, and (11) extension 15° and right bending 15°. First, the 3D pelvic bony model (21) with the IMU sensor was set to the given posture among the 11 postures using commercial CAD software. The images were then taken by projecting the 3D model from frontal and left sagittal views. Thus, the exact values of $\alpha_{Lat}$, $\alpha_{Long}$, $\beta_{Lat}$, and $\beta_{Long}$ in a given posture were known. Then, the measurement errors in $\alpha_{Lat}$, $\alpha_{Long}$, $\beta_{Lat}$, and $\beta_{Long}$ were analyzed by comparing the values obtained from manually measured four points in the IMU sensor and the known exact values. Moreover, the errors between values obtained from manually measured three anatomical points and the known exact values were also compared. The average errors of all participants' results from five trials were analyzed. The interclass correlation coefficients (ICCs) among 10 participants for $\alpha_{Lat}$, $\alpha_{Long}$, $\beta_{Lat}$, and $\beta_{Long}$ were investigated in each measurement cases, using four points in the IMU sensor and using three anatomical points.

## Results

Table 1 shows the average and standard deviation (SD) of errors in $\alpha_{Lat}$, $\alpha_{Long}$, $\beta_{Lat}$, and $\beta_{Long}$ of 11 image sets for each participant when the error was calculated from three anatomical points. Additionally, the average ± SD of 10 averages were provided as 0.6° ± 0.2°, 0.5° ± 0.2°, 0.3° ± 0.1°, and 0.5° ± 0.2°, while the average ± SD of 10 SDs were 0.3° ± 0.1°, 0.5° ± 0.2°, 0.2° ± 0.1°, and 0.5° ± 0.2° in $\alpha_{Lat}$, $\alpha_{Long}$, $\beta_{Lat}$, and $\beta_{Long}$. All averages and SDs were less than 1°. The ICCs among 10 participants for $\alpha_{Lat}$, $\alpha_{Long}$, $\beta_{Lat}$, and $\beta_{Long}$ were 0.9954, 0.9058, 0.9967, and 0.9979, respectively.

**Table 1:** Errors from manually measured three anatomical points (unit: °).

Participant	Error in $\alpha_{Lat}$		Error in $\alpha_{Long}$		Error in $\beta_{Lat}$		Error in $\beta_{Long}$	
	Average	SD	Average	SD	Average	SD	Average	SD
1	0.3	0.2	0.3	0.3	0.2	0.1	0.3	0.4
2	0.8	0.4	0.8	0.6	0.4	0.3	0.7	0.6
3	0.7	0.5	0.5	0.4	0.3	0.2	0.5	0.4
4	0.7	0.3	0.7	0.7	0.3	0.3	0.7	0.7
5	0.5	0.3	0.5	0.4	0.3	0.3	0.4	0.4
6	0.7	0.4	0.7	0.8	0.5	0.5	0.6	0.8
7	0.5	0.2	0.4	0.5	0.3	0.2	0.4	0.5
8	0.7	0.3	0.5	0.4	0.3	0.2	0.5	0.4
9	0.6	0.2	0.4	0.3	0.2	0.1	0.4	0.3
10	0.4	0.1	0.3	0.3	0.2	0.1	0.3	0.3
Average ± SD	0.6 ± 0.2	0.3 ± 0.1	0.5 ± 0.2	0.5 ± 0.2	0.3 ± 0.1	0.2 ± 0.1	0.5 ± 0.2	0.5 ± 0.2

Table 2 shows the average and SD of errors in $\alpha_{Lat}$, $\alpha_{Long}$, $\beta_{Lat}$, and $\beta_{Long}$ of 11 image sets for each participant when using four points in the IMU sensor. The average ± SD of 10 averages were also presented as 3.4° ± 0.3°, 2.0° ± 1.0°, 1.6° ± 0.4°, and 2.1° ± 0.8°, while the average ± SD of 10 SDs were 0.9° ± 0.4°, 1.1° ± 0.4°, 0.6° ± 0.2°, and 1.1° ± 0.4° in $\alpha_{Lat}$, $\alpha_{Long}$, $\beta_{Lat}$, and $\beta_{Long}$. The maximum average and SD of errors were 4.0° in $\alpha_{Lat}$ for participant 6 and 2.0° in $\alpha_{Long}$ for participant 2. The ICCs among 10 participants for $\alpha_{Lat}$, $\alpha_{Long}$, $\beta_{Lat}$, and $\beta_{Long}$ were 0.9933, 0.9668, 0.9933, and 0.9671, respectively.

**Table 2:** Errors from manually measured four points in the IMU sensor (unit: °).

Participant	Error in $\alpha_{Lat}$		Error in $\alpha_{Long}$		Error in $\beta_{Lat}$		Error in $\beta_{Long}$	
	Average	SD	Average	SD	Average	SD	Average	SD
1	3.3	0.6	1.0	0.6	1.3	0.3	1.2	0.7
2	3.7	1.4	3.1	2.0	1.6	0.7	3.0	1.6
3	3.3	0.3	1.7	1.0	1.7	1.0	1.9	1.1
4	3.8	1.0	3.2	1.4	2.2	1.1	3.2	1.8
5	3.3	0.9	1.9	1.0	1.7	0.6	1.9	1.0
6	4.0	1.6	3.8	1.4	2.5	0.7	3.7	1.7
7	3.0	0.8	1.4	1.1	1.3	0.4	1.7	0.9
8	3.6	1.2	1.6	0.7	1.6	0.5	1.6	0.7
9	3.3	0.7	1.2	0.7	1.1	0.3	1.4	0.6
10	3.1	0.5	1.2	1.0	1.4	0.4	1.6	0.8
Average ± SD	3.4 ± 0.3	0.9 ± 0.4	2.0 ± 1.0	1.1 ± 0.4	1.6 ± 0.4	0.6 ± 0.2	2.1 ± 0.8	1.1 ± 0.4

## Discussion

In measuring from three anatomical points, the average errors in $\alpha_{Lat}$, $\alpha_{Long}$, $\beta_{Lat}$, and $\beta_{Long}$ were quite small (less than 0.6°). The SDs for the image set and the participant, which were related to the variability according to the image set and the participant, were also very small (less than 0.3° and 0.5°). Moreover, the ICCs for four angles were greater than 0.9. These results indicate that the measurement of the APP direction using three anatomical points could be considered accurate; thus, it can be used as the true value of APP direction due to the difficulty in the direct measurement of the APP direction in a common clinical setting.

In measuring from four points in the IMU sensor, the average errors in $\alpha_{Lat}$, $\alpha_{Long}$, $\beta_{Lat}$, and $\beta_{Long}$ were acceptable (1.6°–3.4°) in comparison with errors or variations (2°–10°) provided in previous studies (2, 19, 22). Lewinnek et al. proposed 10° of margin in inclination and an anteversion of cup position in THA as a safe zone (2). Wang et al. demonstrated seventy-five percent of the errors across all measurements were within 5° of the radiograph measurements (19). Kalteis et al. showed that the precision of acetabular cup inclination and anteversion were 3° and 10° with plain X-rays, while those were approximately 2° with CT-scan (22). In addition, the SDs for the image set and the participant, which were related to the variability according to the image set and the participant, were also very small (less than 1.4° and 1.1°). Similar to the measurement from three anatomical points, the ICCs for four angles were greater than 0.9. These results also indicate that the measurement of APP direction using four points in the IMU sensor could be considered reliable in terms of intra-participant and inter-participant.

The technique presented in this study, the registration of the IMU sensor position and the APP direction determined from three anatomical points and four IMU points by taking X-ray images from the frontal and sagittal directions, can be applied to measure the APP direction during dynamic motion when the IMU position is obtained from the IMU sensor data instead of the four-point location information. In future study, the registration of the IMU position from four points and the IMU position data from the three inside sensors (accelerometers, gyroscopes, and magnetometer) can be completely obtained by calculating the orthonormal matrix from the local coordinate system in the IMU sensor position to that in the IMU sensor data. Then, the APP direction can be predicted from the IMU sensor data.

There were limitations in this study. Various anatomical and biomechanical factors, which could generate additional error such as the soft tissue tension, contracture, and skin movement as well as the body mass index and pelvic deformation, should be considered in order to enhance the clinical relevance. In addition, more participants can improve statistical confidence since the sample size of study (10 participants) was relatively small.

## Conclusion

This study provided the 3D APP orientation measurement technique by registration with an IMU, and its reliability was tested. The measurement errors in the APP orientation were analyzed by comparing the values obtained from manually measured four points in the IMU sensor and the known exact values in different postures. Moreover, the errors between values obtained from manually measured three anatomical points and the known exact values were also compared. The average errors were quite small when measuring from three anatomical points and were acceptable when measuring from four points in the IMU sensor. The ICCs among participants were greater than 0.9 in both measurements. These results indicate that the measurement of APP direction using four points in the IMU sensor could be considered reliable in terms of intra-participant and inter-participant. The present technique to register the IMU sensor position and the APP direction by taking X-ray images from the frontal and sagittal directions can be fundamental information to measure the APP direction during dynamic motion when the IMU position is obtained from the IMU sensor data instead of the four-point location information.

## Data Availability

The raw data supporting the conclusions of this article will be made available by the authors, without undue reservation.

## References

[1] Ferguson RJ Palmer AJ Taylor A Porter ML Malchau H Glyn-Jones S. Hip replacement. Lancet. (2018) 392(10158):1662–71. 10.1016/S0140-6736(18)31777-X 30496081

[2] Lewinnek GE Lewis JL Tarr R Compere CL Zimmerman JR. Dislocations after total hip-replacement arthroplasties. J Bone Joint Surg Am. (1978) 60(2):217–20. 10.2106/00004623-197860020-00014 641088

[3] Blondel B Parratte S Tropiano P Pauly V Aubaniac JM Argenson JN. Pelvic tilt measurement before and after total hip arthroplasty. Orthop Traumatol Surg Res. (2009) 95(8):568–72. 10.1016/j.otsr.2009.08.004 19910273

[4] Roussouly P Pinheiro-Franco JL. Biomechanical analysis of the spino-pelvic organization and adaptation in pathology. Eur Spine J. (2011) 20(Supp 5):609–18. 10.1007/s00586-011-1928-x 21809016 PMC3175914

[5] Yang G Li Y Zhang H. The influence of pelvic tilt on the anteversion angle of the acetabular prosthesis. Orthop Surg. (2019) 11(5):762–9. 10.1111/os.12543 31663281 PMC6819173

[6] DiGioia AM Hafez MA Jaramaz B Levison TJ Moody JE. Functional pelvic orientation measured from lateral standing and sitting radiographs. Clin Orthop Relat Res. (2006) 453:272–6. 10.1097/01.blo.0000238862.92356.45 17006364

[7] Nishihara S Sugano N Nishii T Ohzono K Yoshikawa H. Measurements of pelvic flexion angle using three-dimensional computed tomography. Clin Orthop Relat Res. (2003) 411:140–51. 10.1097/01.blo.0000069891.31220.fd 12782869

[8] Lee YS Yoon TR. Error in acetabular socket alignment due to the thick anterior pelvic soft tissues. J Arthroplasty. (2008) 23(5):699–706. 10.1016/j.arth.2007.06.012 18534380

[9] Babisch JW Layher F Amiot LP. The rationale for tilt adjusted acetabular cup navigation. J Bone Joint Surg Am. (2008) 90(2):357–65. 10.2106/JBJS.F.00628 18245596

[10] Fritz B Agten CA Boldt FK Zingg PO Pfirrmann CWA Sutter R. Acetabular coverage differs between standing and supine positions: model-based assessment of low-dose biplanar radiographs and comparison with CT. Eur Radiol. (2019) 29(10):5691–9. 10.1007/s00330-019-06136-5 30903332

[11] Lazennec JY Rousseau MA Rangel A Gorin M Belicourt C Brusson A Pelvis and total hip arthroplasty acetabular component orientations in sitting and standing positions: measurements reproductibility with EOS imaging system versus conventional radiographies. Orthop Traumatol Surg Res. (2011) 97(4):373–80. 10.1016/j.otsr.2011.02.006 21570378

[12] Thelen T Thelen P Demezon H Aunoble S Le Huec J-C. Normative 3D acetabular orientation measurements by the low-dose EOS imaging system in 102 asymptomatic subjects in standing position: analyses by side, gender, pelvic incidence and reproducibility. Orthop Traumatol Surg Res. (2017) 103(2):209–15. 10.1016/j.otsr.2016.11.010 28025151

[13] Beangea KHE Chan ADC Beaudette SM Graham RB. Concurrent validity of a wearable IMU for objective assessments of functional movement quality and control of the lumbar spine. J Biomech. (2019) 97(3):109356. 10.1016/j.jbiomech.2019.109356 31668717

[14] Wong WY Wong MS. Trunk posture monitoring with inertial sensors. Eur Spine J. (2008) 17(5):743–53. 10.1007/s00586-008-0586-0 18196296 PMC2367407

[15] Voinea G-D Butnariu S Mogan G. Measurement and geometric modelling of human spine posture for medical rehabilitation purposes using a wearable monitoring system based on inertial sensors. Sensors. (2016) 17(1):3. 10.3390/s17010003 28025480 PMC5298576

[16] Stollenwerk K Müller J Hinkenjann A Krüger B. Analyzing spinal shape changes during posture training using a wearable device. Sensors. (2019) 19(16):3625. 10.3390/s19163625 31434320 PMC6721329

[17] Khuyagbaatar B Purevsuren T Kim YH. Kinematic determinants of performance parameters during golf swing. Proc Inst Mech Eng H. (2019) 233(5):554–61. 10.1177/0954411919838643 30912691

[18] Wada T Nagahara R Gleadhill S Ishizuka T Ohnuma H Ohgi Y. Measurement of pelvic orientation angles during sprinting using a single inertial sensor. Proc AMIA Annu Fall Symp. (2020) 49:10. 10.3390/proceedings2020049010

[19] Wang X Qureshi A Vepa A Rahman U Palit A Williams MA A sensor-based screening tool for identifying high pelvic mobility in patients due to undergo total hip arthroplasty. Sensors. (2020) 20(21):6182. 10.3390/s20216182 33143034 PMC7663251

[20] Stewart J Clegg D Watson S. Calculus. 9th ed. Boston: Cengage Learning (2020).

[21] Khurelbaatar T Kim K Kim YH. A cervico-thoraco-lumbar multibody dynamic model for the estimation of joint loads and muscle forces. J Biomech Eng. (2015) 137(11):111001. 10.1115/1.4031351 26292160

[22] Kalteis TA Handel M Herbst B Grifka J Renkawitz T. In vitro investigation of the influence of pelvic tilt on acetabular cup alignment. J Arthroplasty. (2009) 24(1):152–7. 10.1016/j.arth.2007.12.014 18977116

